# Supplementary material for: Inelastic electron scattering induced quantum coherence in molecular dynamics
Source: Nat Commun. 2023 May 13;14:2769. doi: 10.1038/s41467-023-38440-6 (PMC10183011; doi:10.1038/s41467-023-38440-6)
Supplement: Supplementary file 1 — Suplementary information [file 41467_2023_38440_MOESM1_ESM.pdf]

SUPPLEMENTARY INFORMATION

Inelastic electron scattering induced quantum coherence in molecular dynamics

Akshay Kumar[1], Suvasis Swain[1, #], and Vaibhav S. Prabhudesai[1, *]

[1]*Tata Institute of Fundamental Research, Colaba, Mumbai 400005 India*

[#]*Current Affiliation: Centre de Recherche sur les Ions, les Matériaux et la Photonique (CIMAP) - UMR 6252 - Normandie Université, ENSICAEN, UNICAEN, CEA, CNRS, 14000 Caen, France*

[*]vaibhav@tifr.res.in

Supplementary Note 1: Velocity slice images used for measuring the forward-backward asymmetry at different electron energies.

The forward-backward asymmetry shown in figure 3 of the main text at various electron energies is calculated using the VSI images of the H^- ions produced by the ion-pair formation. The images are shown in supplementary figure 1. These momentum images are obtained by adding about 5000 to 10000 captured images (each with 10 sec exposure time on the CCD camera) for the static gas and crossed beam geometry each and then subtracting them. The number of images varied based on the cross-section of the process.

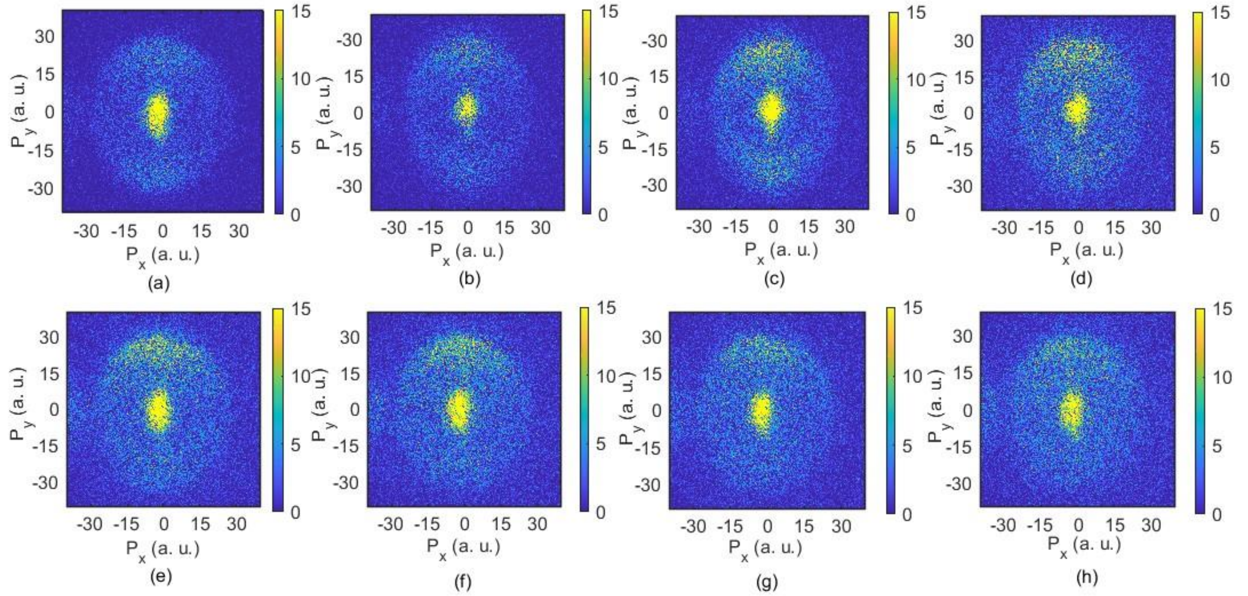

Supplementary Fig. 1 The momentum images obtained for the ion-pair formation process in H_2 at (a - h) 30eV, 35 eV, 40 eV, 60 eV, 70 eV, 80 eV, 90 eV, and 100 eV electron energies, respectively
